# Microvascular Structure Changes After Intravitreal Ranibizumab Injection in Retinal Vein Occlusion Patients With and Without Macular Ischemia

**DOI:** 10.3389/fmed.2021.737537

**Published:** 2021-11-12

**Authors:** Ziyi Zhu, Yongan Meng, Igor Kozak, Manyun Xie, Youling Liang, Bin Yan, Liang Zhou, Pingbo Ouyang, Xiaoxi Yao, Jing Luo

**Affiliations:** ^1^Department of Ophthalmology, The Second Xiangya Hospital, Central South University, Changsha, China; ^2^Hunan Clinical Research Center of Ophthalmic Disease, Changsha, China; ^3^Moorfields Eye Hospital UAE, Abu Dhabi, United Arab Emirates; ^4^Shenzhen College of International Education, Shenzhen, China

**Keywords:** anti-VEGF treatment, macular ischemia, OCTA, ischemic index, RVO, vessel density (VD)

## Abstract

**Purpose:** To investigate the changes in the macular microvascular structure after anti-vascular endothelial growth factor (anti-VEGF) treatment in retinal vein occlusion (RVO) patients with and without macular ischemia.

**Methods:** A total of 39 patients were divided into the macular ischemia group (*n* = 22) and the nonischemia group (*n* = 17) at baseline. All the patients received an intravitreal injection of ranibizumab with a 3+ pro re nata (PRN) regimen. The foveal avascular zone (FAZ) areas, macular vessel density (VD), and macular ischemic index (ISI) were evaluated at baseline and 3 and 6 months after treatment.

**Results:** After treatment, some patients in the macular ischemia group achieved obvious reperfusion in macular nonperfusion areas. The VD and macular ISI improved in RVO patients, but the changes in VD and macular ISI were different in the two groups. The improvement of best corrected visual acuity (BCVA) was positively correlated with the improvement of macular perfusion status. Macular perfusion remained stable in most patients in RVO and only one patient had macular ischemia aggravation.

**Conclusion:** The macular microvascular structures were stable in most RVO patients after anti-VEGF treatment. At the same time, some patients with macular ischemia presented reperfusion in macular nonperfusion areas, and still a few patients presented aggravated macular ischemia. Macular ISI is a good way to evaluate macular perfusion status in RVO compared to VD.

## Introduction

Retinal ischemia caused by retinal vein occlusion (RVO) has been of great concern in clinical studies and is present in ~66% of main branch RVO (BRVO) and ischemic central RVO (CRVO) cases (1, 2). Anti-vascular endothelial growth factor (VEGF) treatment, as a first-line treatment for RVO, has been proven to significantly improve macular edema. However, it has been controversial whether anti-VEGF treatment aggravates ischemia. Terui et al. (3) reported increased nonperfusion areas after anti-VEGF treatment in several cases. However, other studies have suggested that retinal ischemia develops gradually and irreversibly, irrespective of anti-VEGF treatment (4, 5). Campochiaro et al. (6) have shown that anti-VEGF treatment not only effectively improves macular edema but also prevents the progression of nonperfusion areas. In contrast, some researchers still believe that no reperfusion occurs after anti-VEGF treatment, which may be due to limited sample sizes and short observation periods; the number of patients was 9 and 14, and the observation time was only 3 months in those studies (7, 8). Moreover, no study has compared the different anti-VEGF treatments in RVO patients with and without macular ischemia.

Thus, we aimed to investigate the effects of ranibizumab with a 3+ pro re nata (PRN) regimen on macular perfusion status in RVO patients with and without macular ischemia. We wished to analyze the changes in macular perfusion and focus on whether the macular nonperfusion (MNP) area improved after anti-VEGF treatment, especially in RVO patients with macular ischemia. Optical coherence tomography angiography (OCTA) measurement values have been used as biomarkers in monitoring disease progression and treatment response in many clinical studies (9). Therefore, we quantified foveal avascular zone (FAZ) areas, capillary density, and macular ischemic index (ISI) in the macular region by using OCTA.

## Materials and Methods

### Patients

This study followed the tenets of the Declaration of Helsinki. All the patients gave informed written consent before participating in this study. All the procedures were performed in accordance with the Ethical Standards of the Second Xiangya Hospital of Central South University Committee and the ethical review approval number was 2017-053. The study period was between September 2018 and December 2019 and all the patients were diagnosed with RVO. Only one eye of each patient was included in the study. All the eyes were injected with 0.3 mg of ranibizumab at monthly intervals for the first 3 months and given additional interval injections according to the PRN regimen. All the patients were evaluated for 6 months. Before the first intraocular injection, all the patients underwent fluorescein angiography (FA) and OCTA, which showed whether a patient had apparent ischemia and nonperfusion areas. The exclusion criteria were poor OCTA image quality before grouping patients, Q-score below 7, the opacity of refractive media, the presence of significant residual motion artifacts, and severely disrupted anatomical features of the macular area (such as severe cystoid macular edema) leading to the segmentation errors. Then, the patients were divided into two groups, namely, the macular ischemia group and the nonischemia group. The macular ischemia group was defined as the presence of retinal capillary loss or nonperfusion in the Early Treatment Diabetic Retinopathy Study (ETDRS) grid center subfield according to the CRYSTAL study (10). Additional exclusion criteria included patients with retinal arterial occlusion; retinal treatment or major ocular surgery within the prior 6 months; vision loss caused by any other retinal disease, including severe diabetic retinopathy and age-related macular degeneration (AMD); intraocular pressure ≥ 25 mm Hg; refractive error (myopia > 6 D or hyperopia > 3D); poor refractive media; and other systemic diseases that required hospitalization.

Ranibizumab (RBZ, Lucentis; Genentech Incorporation, South San Francisco, California, USA) is a monoclonal antibody against VEGF-A that effectively reduces VEGF concentrations by blocking VEGF binding to VEGF receptors (11). Some large multicenter clinical trials, including the Treatment of Macular Edema following Branch Retinal Vein Occlusion: Evaluation of Efficacy and Safety (BRAVO) and the Treatment of Macular Edema following Central Retinal Vein Occlusion: Evaluation of Efficacy and Safety (CRUISE) studies, have proven that ranibizumab effectively improved visual acuity (VA) and macular edema and affected retinal nonperfusion (12, 13).

### Image Acquisition and Data Measurement

All the OCTA images were acquired by the AngioVue OCT System version 2018.0.0.14 (RTVue XR Avanti, Optovue Incorporation, Fremont, California, USA) by using the split-spectrum amplitude decorrelation angiography (SSADA) algorithm to detect blood flow and provided a new method for rapid imaging of detailed microvasculature at the distinct depths (14). The system reduced artifacts by motion correction technology (MCT) and three-dimensional (3D) projection artifact removal (PAR) to remove artifacts and PAR differentiated *in-situ* OCTA signals from the projection artifacts and removed the projection artifacts (14). AngioVue provided an automated software algorithm to generate the boundaries of the superficial capillary plexus (SCP) (from the internal limiting membrane (ILM) to 10 μm above the inner plexiform layer (IPL)) and deep capillary plexus (DCP) (from 10 μm above the IPL to 10 μm below the outer plexiform layer(OPL)). Additionally, the boundary of the FAZ area was from the ILM to 10 μm below the OPL, while the segmentation boundaries could also be adjusted and corrected by manual segmentation (15). Parameters of blood flow status in the macular region are derived from a 6 × 6 mm macular scan grid instead of a traditional 3 × 3 mm macular scan grid. This provides a new method to analyze the changes in macular ischemia after anti-VEGF treatment. The central foveal thickness (CFT), as an average value within a circular 1 mm diameter area centered in the fovea, was automatically measured by OCTA. The values of the FAZ area and vessel density (VD) were also automatically calculated by OCTA software when the segmentation boundaries were corrected. Tsui et al. (16) proposed the concept of ISI and provided us with a new way to evaluate the ischemic state in retinal vascular diseases by calculating areas of ischemia as a percentage of the total calculated visible retina in ultrawide-field angiography (UWFA) images. We similarly applied the macular ISI concept to analyze macular ischemia status by using OCTA. Macular ISI was calculated by areas of nonperfusion area as a percentage of the total calculated area. The calculation formula is as follows:


$$\begin{array}{l} \;Macular\;ISI\\ =\frac{Nonperfusion\;area}{Measurement\;area\;\left(6\;mm\;\times \;6\;mm\;diameter\;circle\;area\right)}\\ \times 100\%=\frac{Measurement\;area-Perfusion\;area-FAZ\;area}{Measurement\;area}\\ \;\times 100\%=\left(1-\frac{Perfusion\;area+FAZ\;area}{Measurement\;area}\right)\\ \times 100\%=\left(1-\frac{\frac{VD}{100\%}\;\times \;Measurement\;area+FAZ\;area}{Measurement\;area}\right)\\ \;\times 100\%\\ Measurement\;area=3\;mm\times 3\;mm\times \pi =28.27\;mm2\\ \;Macular\;ISI=\left(1-\frac{VD}{100\%}-\frac{FAZ\;area}{28.27}\right)\times 100\% \end{array}$$

**Figure 1:**
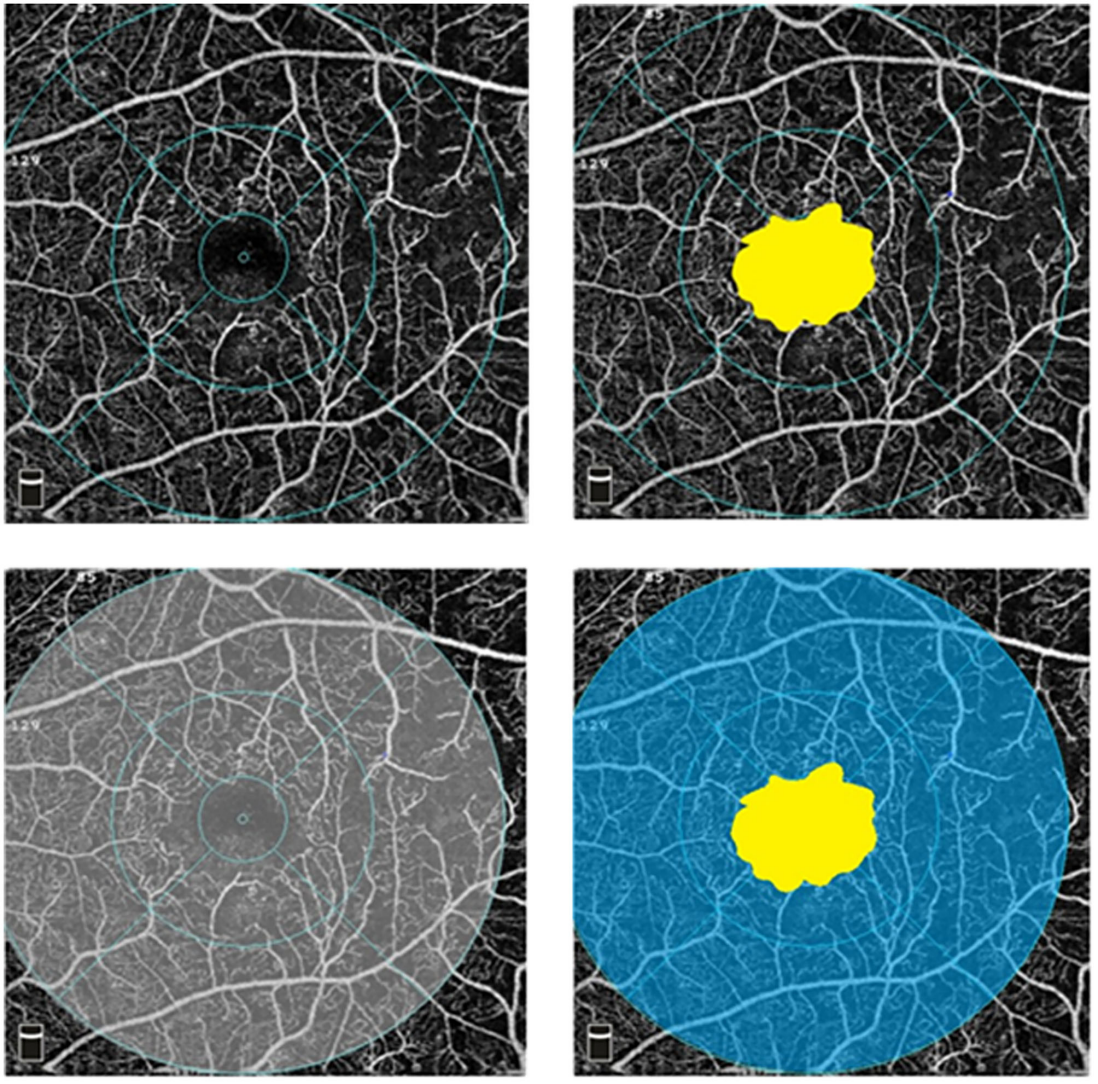
Optical coherence tomography angiography (OCTA) images in superficial capillary plexus (6 × 6 mm). The foveal avascular zone (FAZ) area measurement is located in the yellow area, vessel density (VD) measurement is located in the gray area, and ischemic index (ISI) measurement is located in the blue area.

An example is shown in Figure 1. The FAZ area measurement is located in the yellow area, VD measurement is located in the gray area, and macular ISI measurement is located in the blue area. The macular ISI mainly excludes the influence of changes in the FAZ area and more intuitively represents the ischemic state in the macular region with a larger macular ISI indicating more severe ischemia and non-perfusion.

### Statistical Analysis

The main measurements, including best corrected visual acuity (BCVA), CFT, FAZ area, superficial and deep VD, and macular ISI in this study, were tested by the Kolmogorov–Smirnov test (*p* > 0.05 was set as statistically significant). At 0, 3, and 6 months, changes in FAZ area, blood flow, and macular ISI were evaluated by the paired sample *t*-test. Moreover, *p* < 0.05 indicated statistical significant. Statistical analysis was performed by using the Statistical Package for the Social Sciences (SPSS) version 25.0 (SPSS Incorporation, Chicago, Illinois, USA).

## Results

We enrolled 39 patients with RVO in two groups: the macular ischemia group (*n* = 22) and the nonischemia group (*n* = 17). The characteristics of the patients at baseline are shown in Table 1. The patients in the macular ischemia group showed ischemic changes, such as FAZ area expansion, MNP, decrease in blood flow density, and increase in macular ISI, especially in the superficial layer.

**Table 1:** Characteristics of the macular ischemia group and the nonischemia group at baseline.

		**Macular ischemia group (*n* = 22)**	**Nonischemia group (*n* = 17)**	**Total (*n* = 39)**
Gender (male:female)		9:13	11:6	20:19
CRVO:BRVO		7:15	7:10	14:25
Age (years)		57.8 ± 7.6	56.4 ± 8.0	57.2 ± 7.7
BCVA (logMAR)		0.85 ± 0.42	0.54 ± 0.37	0.71 ± 0.42
CFT (μm)		324 ± 128	398 ± 152	357 ± 142
IOP (mmHg)		16.0 ± 2.2	16.9 ± 2.2	16.4 ± 2.2
FAZ area (mm^2^)		0.463 ± 0.249	0.351 ± 0.130	0.414 ± 0.210
VD (%)	SCP	40.2 ± 4.6	48.0 ± 3.0	43.6 ± 5.5
	DCP	41.3 ± 4.4	45.4 ± 3.6	43.1 ± 4.5
ISI (%)	SCP	57.8 ± 4.5	50.6 ± 2.9	54.6 ± 5.3
	DCP	56.9 ± 4.6	53.3 ± 3.8	55.3 ± 4.6

*BCVA, best corrected visual acuity; CFT, central foveal thickness; IOP, intraocular pressure; FAZ, foveal avascular zone; VD, vessel density; ISI, ischemic index; SCP, superficial capillary plexus*.

**Figure 2:**
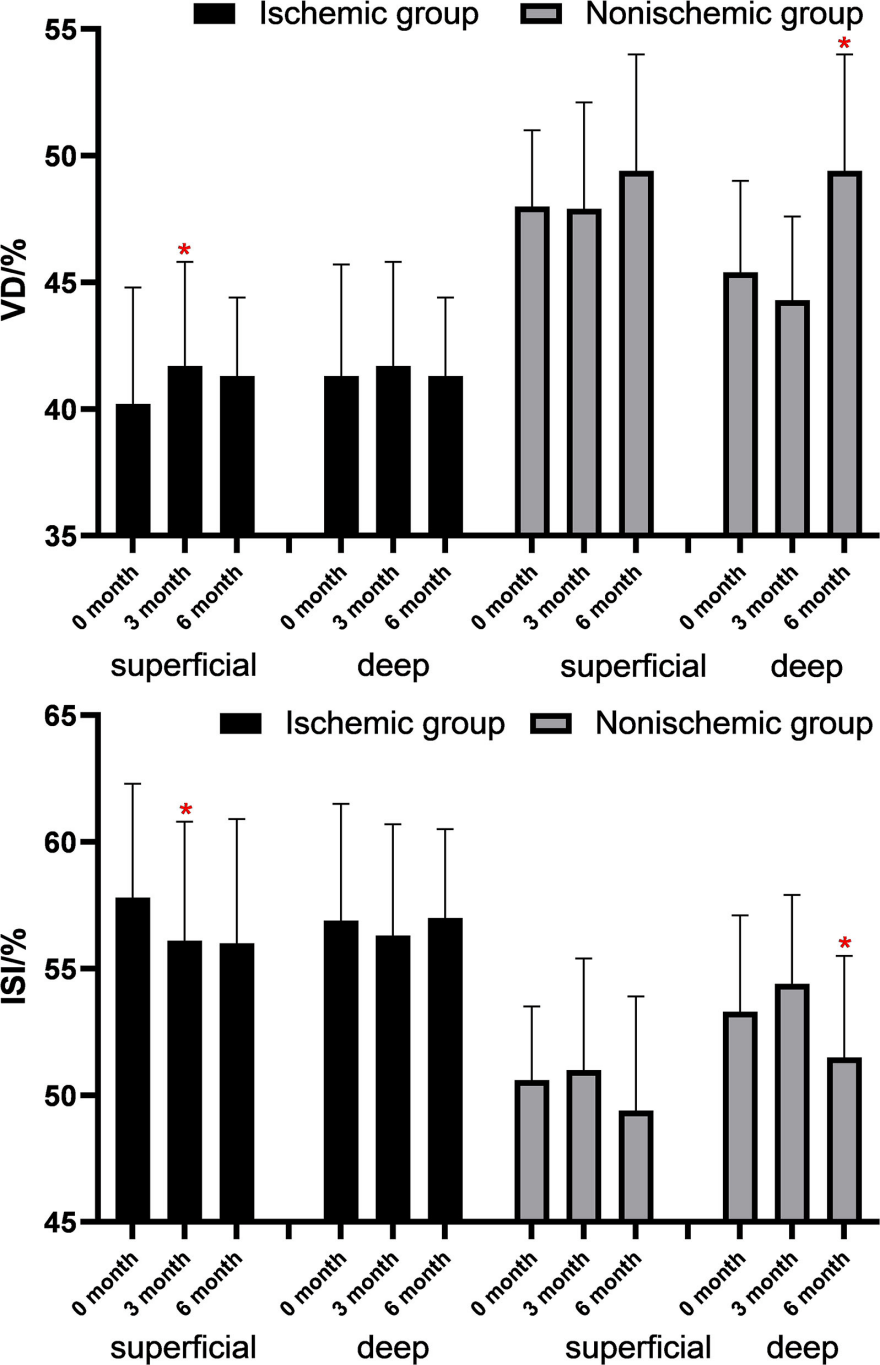
Changes of VD and ISI in the macular ischemia group after antivascular endothelial growth factor (VEGF) treatment. *p < 0.05.

The changes in FAZ area, CFT, VD, and macular ISI are listed in Table 2 and the changes in the macular perfusion status in the macular ischemia group are depicted in Figure 2. In the macular ischemia group, the logarithm of the minimum angle of resolution (logMAR) BCVA changed from 0.85 ± 0.42 to 0.61 ± 0.35 (*p* < 0.001) and then to 0.60 ± 0.41 (*p* = 0.908) and the FAZ area and CFT showed no significant changes. The superficial layer VD and macular ISI changed significantly at 3 months and remained stable until the endpoint. The superficial layer VD changed from 40.2 ± 4.6% to 41.7 ± 4.8% (*p* = 0.009) and then to 41.7 ± 5.2% (*p* = 0.966), whereas the superficial macular ISI changed from 57.8 ± 4.5% to 56.1 ± 4.7% (*p* = 0.010) and then to 56.0 ± 4.9% (*p* = 0.902) at the endpoint. Moreover, the VD and macular ISI remained stable during the study period in the deep layer. In the nonischemia group, the logMAR BCVA changed from 0.54 ± 0.37 to 0.36 ± 0.30 (*p* = 0.008) and then to 0.29 ± 0.24 (*p* = 0.533). The deep layer VD changed from 45.4 ± 3.6 to 44.3 ± 3.3% (*p* = 0.135) and then to 47.4 ± 3.9% (*p* = 0.023), whereas the deep macular ISI changed from 53.3 ± 3.8 to 54.4 ± 3.5% (*p* = 0.150) and then to 51.5 ± 4.0% (*p* = 0.030) at the endpoint.

**Table 2:** Changes in the BCVA, CFT, FAZ area, VD, and macular ISI between the macular ischemia group and the nonischemia group.

	**BCVA/logMAR**	**CFT/μm**	**FAZ area/mm^2^**	**VD**		**ISI**	
				**Superficial-VD/%**	**Deep-VD/%**	**Superficial-ISI/%**	**Deep-ISI/%**
**Macular ischemia group**							
Month 0	0.85 ± 0.42	324 ± 128	0.463 ± 0.249	40.2 ± 4.6	41.3 ± 4.4	57.8 ± 4.5	56.9 ± 4.6
Month 3	0.61 ± 0.35^*^	293 ± 98	0.447 ± 0.190	41.7 ± 4.8^*^	41.7 ± 4.1	56.1 ± 4.7^*^	56.3 ± 4.4
Month 6	0.60 ± 0.41	280 ± 77	0.465 ± 0.247	41.7 ± 5.2	41.3 ± 3.1	56.0 ± 4.9	57.0 ± 3.5
**Nonischemia group**							
Month 0	0.54 ± 0.37	398 ± 152	0.351 ± 0.130	48.0 ± 3.0	45.4 ± 3.6	50.6 ± 2.9	53.3 ± 3.8
Month 3	0.36 ± 0.30^*^	347 ± 106	0.346 ± 0.126	47.9 ± 4.2	44.3 ± 3.3	51.0 ± 4.4	54.4 ± 3.5
Month 6	0.29 ± 0.24	311 ± 95	0.336 ± 0.110	49.4 ± 4.6	47.4 ± 3.9^*^	49.4 ± 4.5	51.5 ± 4.0^*^

* *p < 0.05*.

*BCVA, best corrected visual acuity; CFT, central foveal thickness; FAZ, foveal avascular zone; VD, vessel density; ISI, ischemic index*.

**Figure 3:**
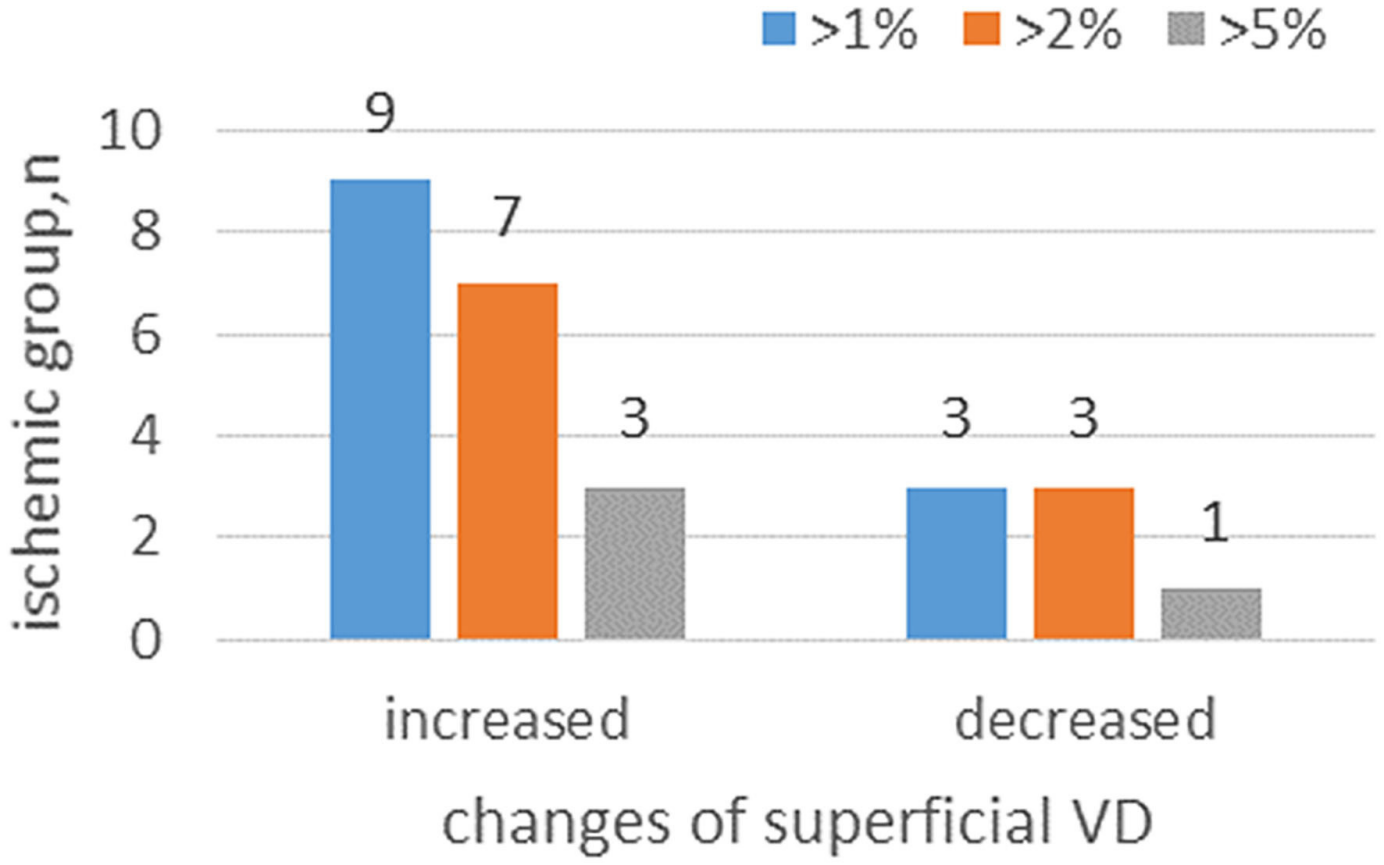
The distribution of the number of patients with VD changes.

At 6 months, from a total of 22 patients with macular ischemia at baseline, nine patients had increased superficial VD by more than 1%, seven patients had increased superficial VD by more than 2%, and three patients had increased superficial VD by more than 5%. Only three patients had decreased superficial VD by more than 1%, three patients had decreased superficial VD by more than 2%, and two patients had decreased superficial VD by more than 5% (Figure 3). Three patients demonstrated reperfusion and only one patient had an enlarged MNP area. The VD and macular ISI remained stable during the study period in the nonischemia group.

**Figure 4:**
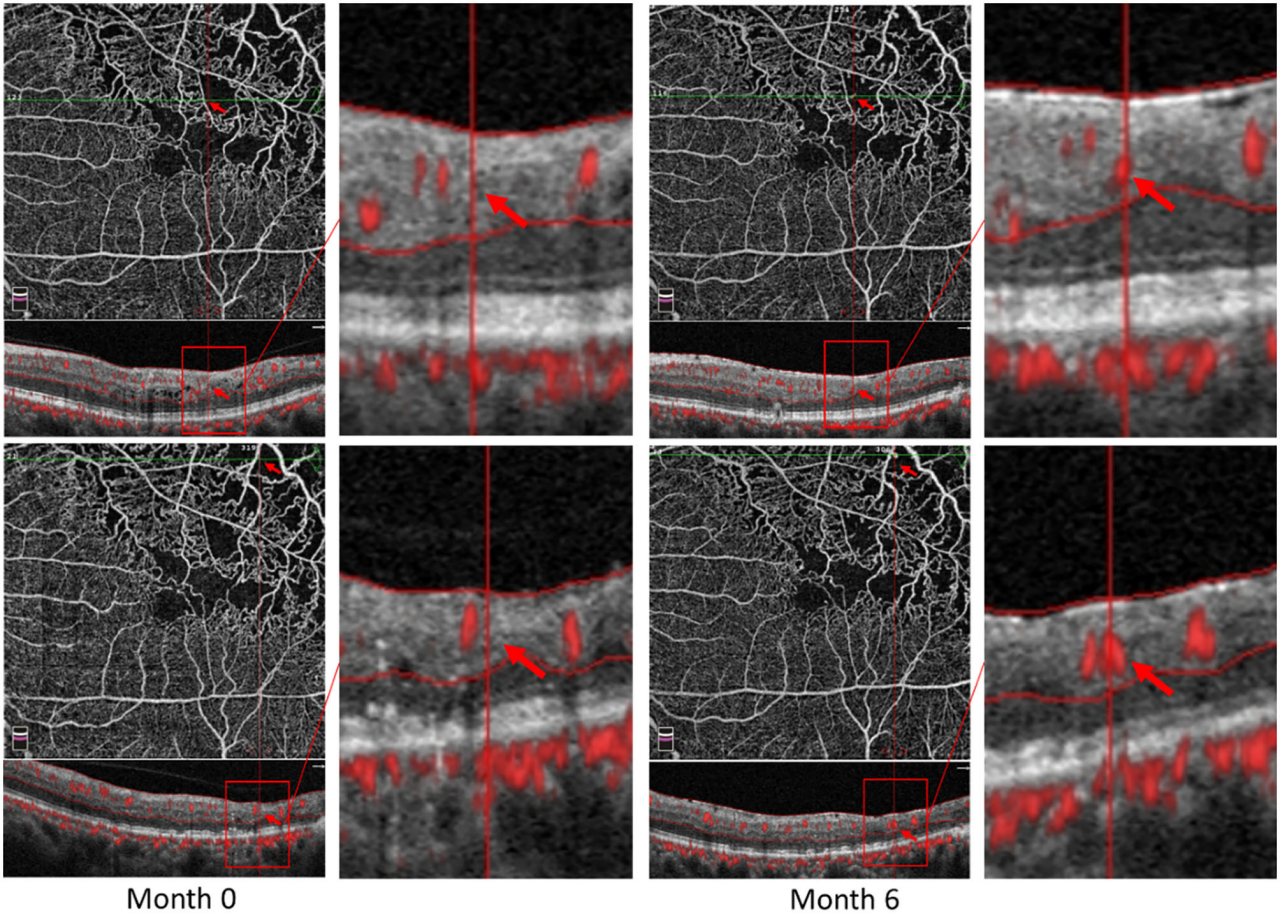
Reperfusion occurred in the left eye of a 56-year-old male with macular ischemia after receiving anti-VEGF treatment. Red arrows indicate the growth of new capillaries and reperfusion in the macular nonperfusion areas.

**Figure 5:**
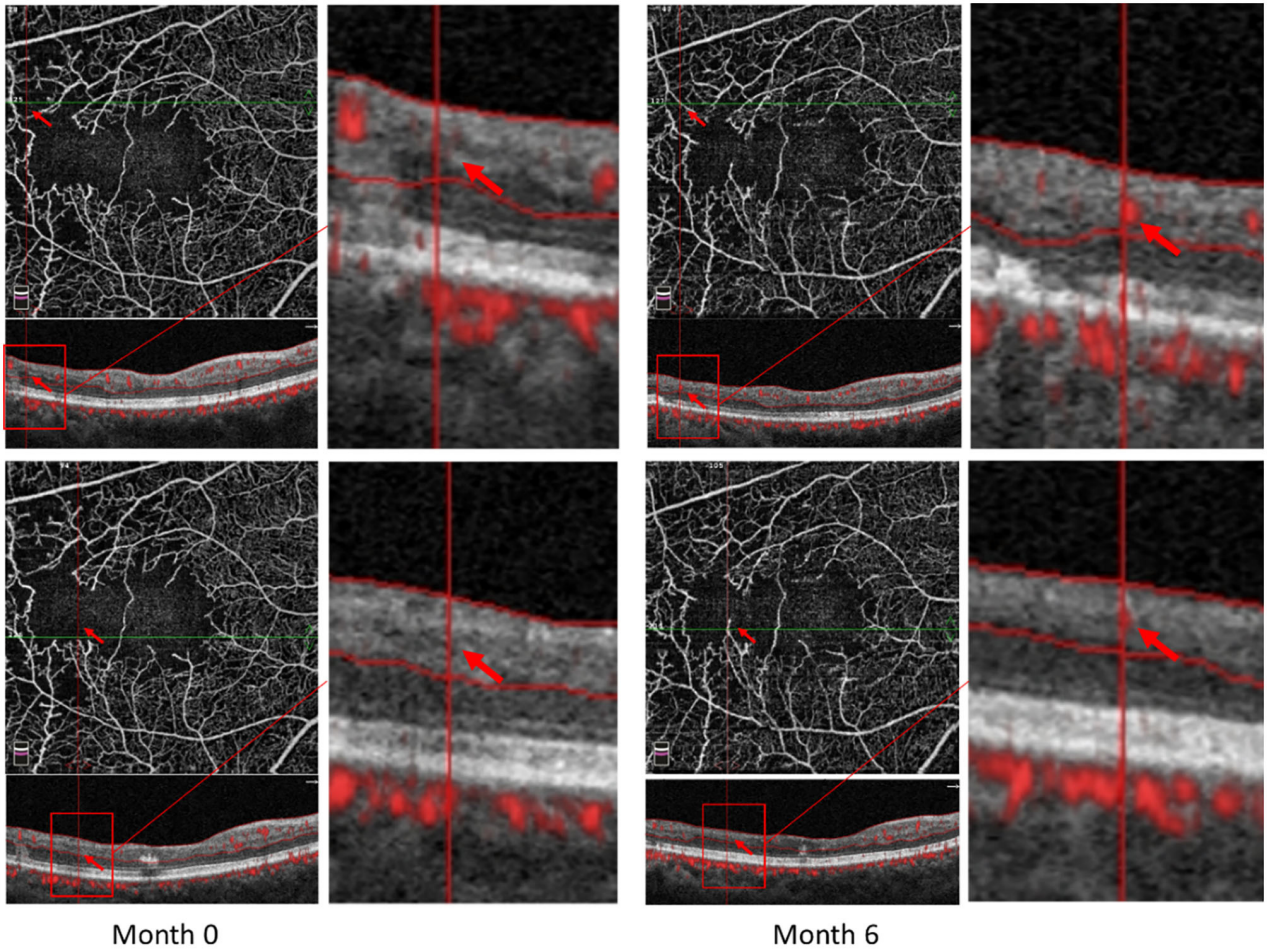
Reperfusion occurred in the right eye of a 57-year-old female with macular ischemia after receiving anti-VEGF treatment. Red arrows indicate the growth of new capillaries and reperfusion in the macular nonperfusion areas.

**Figure 6:**
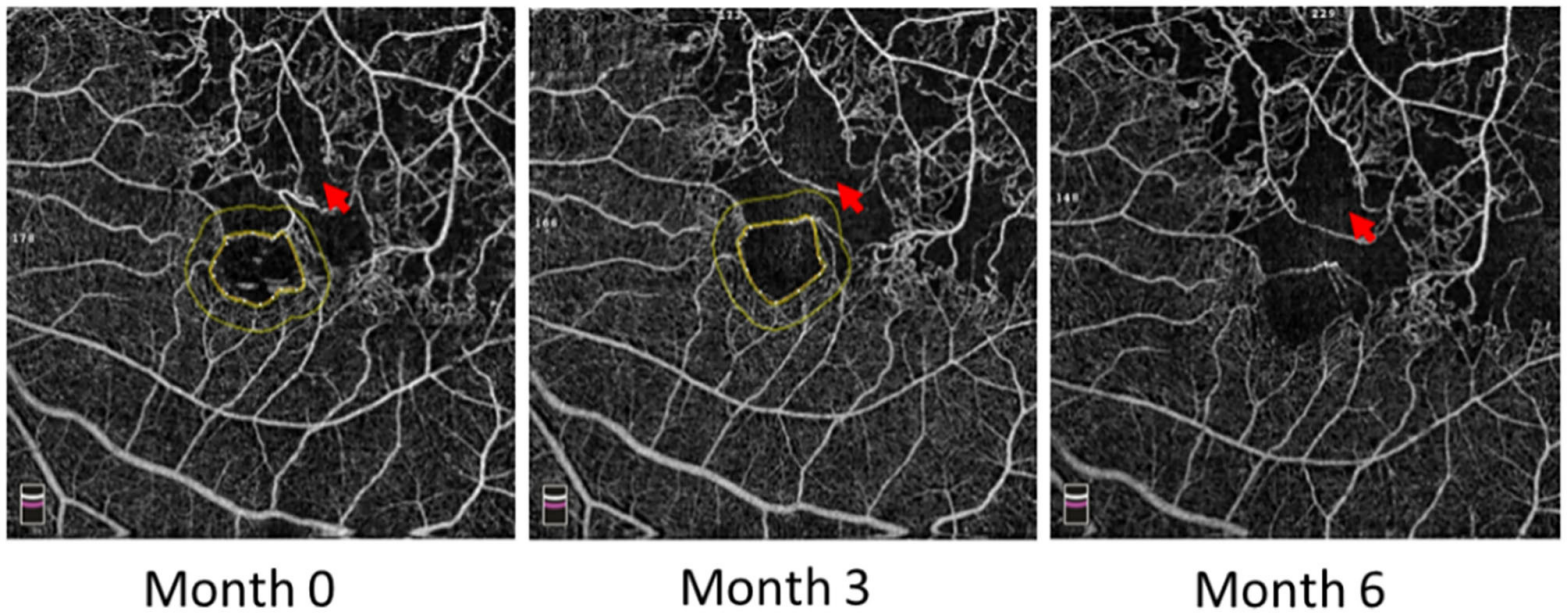
Macular ischemia aggravated in the left eye of a 60-year-old male after receiving anti-VEGF treatment. Red arrows indicate the nonperfusion area gradually increased and macular ischemia aggravated.

Some patients with macular ischemia demonstrated reperfusion of previously nonperfused areas (Figures 4, 5), and the red arrows indicate the growth of new capillaries and reperfusion in the MNP areas. There was still one patient with macular ischemia that was aggravated after receiving treatment (Figure 6) and the red arrows indicate that the nonperfusion area gradually increased and macular ischemia was aggravated.

**Figure 7:**
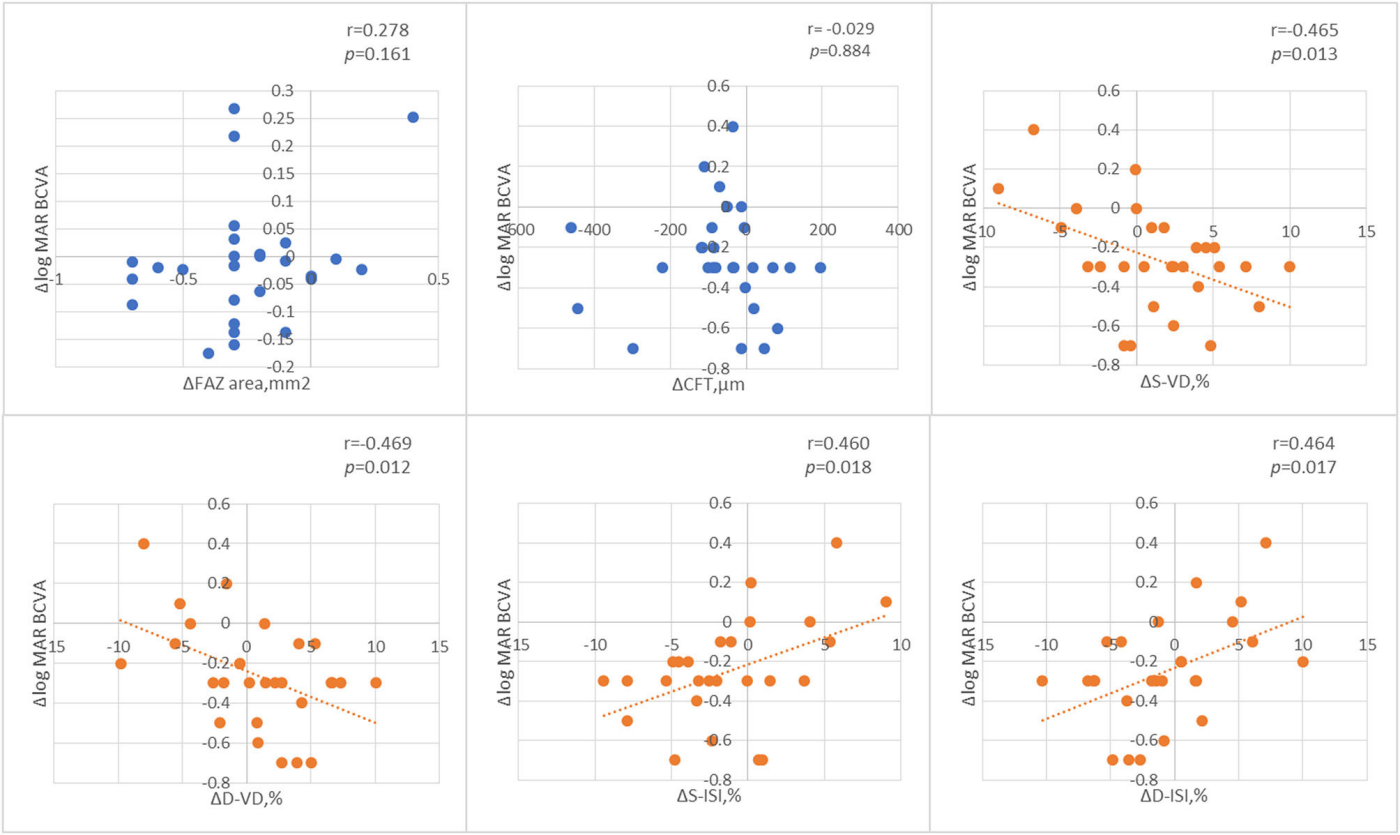
The Pearson correlation analysis of the changes of best corrected visual acuity (BCVA) and macular perfusion indexes.

Figure 7 shows the Pearson correlation analysis of the changes in BCVA and macular perfusion indexes in all the RVO patients. The changes in both the superficial and deep layer of VD were negatively correlated with the changes in the logMAR BCVA and the changes in both the superficial and deep layer macular ISI were positively correlated with the changes in the logMAR BCVA.

## Discussion

Retinal ischemia was the main sign of aggravation of RVO and approximately one-third of nonischemic CRVO converted to ischemic CRVO within 3 years (1, 2). Studies have also demonstrated that the capillary density and morphology have a significant association with the severity of RVO. Furthermore, the baseline BCVA in ischemic eyes was usually worse than that in nonischemic eyes. The extent of ischemia in the macular region was closely associated with the prognosis of BCVA, where patients with greater macular perfusion status were more likely to show a better improvement in macular edema and BCVA (17–19). Thus, it is valuable to evaluate macular perfusion status. At present, most studies use VD to evaluate macular perfusion status; however, the disadvantage is that the changes in VD can be influenced by changes in the FAZ area. Thus, we use macular ISI as an evaluation index. Tsui et al. (16) first proposed the concept of ISI to evaluate the ischemic state by calculating areas of ischemia as a percentage of the total calculated visible retina. We applied the macular ISI concept to analyze macular ischemia status. The concept of macular ISI provides us with a new way to evaluate the ischemic state in macular ischemia status and excludes the influence of changes in the FAZ area; thus, it could more intuitively represent the ischemic state in the macular region. Macular ISI was highly correlated with blood flow density in both the superficial and deep layers. However, when the area of the FAZ changes greatly, macular ISI evaluating the change in macular perfusion status is more accurate than VD.

In this study, we mainly adopted OCTA as the main evaluation method to observe the macular perfusion status. OCTA is highly consistent with FA in showing the vessels in the macular area and is not affected by the leakage of fluorescein. OCTA is noninvasive, repeatable, and easily operable, and it is subtler than FA in displaying macular area capillaries (14). OCTA has obvious advantages in quantifying VD, FAZ areas, and nonflow areas. Previous studies in RVO have also shown that OCTA detected nonperfusion areas and superficial and deep capillary abnormalities more often than FA and OCTA measurements values may serve as biomarkers for monitoring disease progression and treatment response (32).

In OCTA, total VD is divided into SCP and DCP. The status of superficial VD is associated with macular edema development, whereas VD in the deep plexus is important for the oxygen requirements of photoreceptors and the outer retina (20). Researchers suggest that microvascular changes, such as MNP areas of the RVO, are observed more obviously in the deep plexus than in the superficial plexus (21). Deep VD is related to choriocapillaris blood flow and is more susceptible to ischemia.

Overall, this study indicates that anti-VEGF treatment has a positive effect on macular ischemia and improves macular perfusion status in most cases. Our data suggested that the changes in VD and macular ISI were different in the macular ischemia group and the nonischemia group. In the RVO with macular ischemia, macular ischemia decreased significantly after anti-VEGF treatment, especially in the superficial retinal layer, and the VD and macular ISI in the deep plexus remained stable without exacerbation of ischemia. In the nonischemia group, VD and macular ISI still improved in the deep layer at 6 months. In addition, the improvement in BCVA was positively correlated with the improvement in macular perfusion status.

The improvement in VD and macular ISI was different between the macular ischemia group and the nonischemia group. Why is there such a difference between the two groups? Our data showed that the superficial VD in the macular ischemia group decreased more significantly than deep VD and the superficial macular ISI increased more significantly than deep macular ISI at baseline compared with the nonischemic group. The DCP, where the inner nuclear layer, is located maybe highly vulnerable to ischemia because the DCP supplies oxygen to the photoreceptor and upregulates VEGF more strongly than it does in the SCP (22, 23). Therefore, we suspect that in the macular ischemia group, anti-VEGF treatment can improve retinal hypoxia and give priority to oxygen supply to SCP. As SCP did not show severe ischemia and hypoxia, DCP improved in the nonischemia group. This speculation needs to be confirmed in future studies.

Macular perfusion was not aggravated in most patients with RVO. In the macular ischemia group, the number of patients with increased superficial VD was more than the number of patients with decreased superficial VD in 6 months. Notably, some patients even demonstrated reperfusion after anti-VEGF treatment in the nonperfusion areas. Vascular reperfusion should be differentiated from neovascularization and collateral vessels. First, the shape of the reperfused vasculature is almost the same as that of the original vasculature, while neovascularization is usually dilated and tortuous. Second, OCTA also showed that there are no neovascularization or collateral vessels. The development of neovascularization usually appears in the superficial layer and has a tendency to break through the internal limiting membrane (ILM) and grows into the vitreous cavity, while the reperfused vasculature in this study was at a deep layer on OCTA instead of the superficial layer and did not break through the ILM. In addition, collateral vessels usually form in the acute phase in RVO (24). In this study, the reperfusion of RVO occurred more than 3 months after anti-VEGF treatment and OCTA confirmed that it was not collateral circulation.

The mechanism of these changes caused by anti-VEGF treatment is still unclear. Animal experiments have proven that the suppression of VEGF causes reperfusion of the closed vessels (25). Studies have demonstrated that anti-VEGF treatment could not only reduce autophagy and apoptosis rates and activate ischemia-damaged microglia to protect the retinal ganglion cells and bipolar cells, further normalize peripheral cells, stabilize the basement membrane, but also reopen closed retinal vessels, prevent the progression of vessel closure, and improve retinal ischemia (26, 27). Recently, Seo et al. (28) found that anti-VEGF treatment inhibited the upregulation of VEGF in both the superficial and deep capillary plexuses by decreasing the leukocyte aggregation and retinal hyperpermeability.

However, one case in this study was found that macular ischemia was aggravated after anti-VEGF treatment, which is consistent with some early studies. Takayuki et al. observed the deterioration of retinal perfusion status in BRVO, but only a few (1.7%) patients presented a significant increase in nonperfusion areas (NPAs) greater than 1.0 in the disk area (3). However, a natural history of RVO showed that retinal ischemia would develop gradually and irreversibly (3). Retinal ischemia aggravated in RVO patients after anti-VEGF therapy, especially in patients with preexisting retinal ischemia. The Central Vein Occlusion Study (CVOS) indicates the conversion rate from nonischemic RVO to ischemic RVO which is 3.3% by 4 months (29). This study found that only one case (5.9%) had a significant extension of MNP in 6 months and probably reflected a conversion from nonischemia to ischemia in few patients with RVO. Due to the great hemodynamic abnormalities in some RVO patients and retinal capillary endothelial cells in a state of oxidative stress, macular ischemia might be enlarged before macular capillaries completely closed (30, 31).

This study has several limitations. First, we adopted OCTA as the main evaluation method to observe the blood flow of the macular region and to quantify macular ischemia, but fluid may induce segmentation artifacts. Fluorescein Fundus Angiography (FFA) is still considered a good method in retinal imaging, especially, in the observation of changes in the dynamic blood flow and retinal capillary leakage, but it is an invasive method requiring venipuncture and contrast infusion with poor repeatability. Second, the observation period and sample size are relatively short and small, respectively. A larger sample size and a longer observation period will benefit the determination of how anti-VEGF plays a role in improving macular ischemia and retinal ischemia.

In conclusion, macular ISI is a good way to evaluate the macular perfusion status in RVO. The changes in VD and macular ISI were different in the macular ischemia group and the nonischemia group. The macular microvascular structures were stable in most RVO patients after anti-VEGF treatment. At the same time, some patients with macular ischemia presented reperfusion in MNP areas, and still, a few patients presented aggravated macular ischemia. In addition, BCVA was positively correlated with macular perfusion status in RVO.

## Data Availability Statement

The raw data supporting the conclusions of this article will be made available by the authors, without undue reservation.

## Ethics Statement

The studies involving human participants were reviewed and approved by Ethics Committee of the Second Xiangya Hospital of Central South University (Registration Number: 2010 K059). The patients/participants provided their written informed consent to participate in this study. Written informed consent was obtained from the individual(s) for the publication of any potentially identifiable images or data included in this article.

## Author Contributions

ZZ and JL were responsible for the conception and design of this research and wrote the draft. ZZ, YM, YL, BY, and PO acquired the data. ZZ, YM, and XY analyzed and interpreted the data. IK, MX, and LZ revised the manuscript critically. The manuscript benefited from the use of editorial service of American Journal Experts (AJE). All authors have read and approved the final manuscript.

## Funding

This study was supported by the Natural Science Foundation of Hunan Province, China (S2020JJMSXM2986).

## Conflict of Interest

The authors declare that the research was conducted in the absence of any commercial or financial relationships that could be construed as a potential conflict of interest.

## Publisher's Note

All claims expressed in this article are solely those of the authors and do not necessarily represent those of their affiliated organizations, or those of the publisher, the editors and the reviewers. Any product that may be evaluated in this article, or claim that may be made by its manufacturer, is not guaranteed or endorsed by the publisher.
